# Assembling real networks from synthetic and unstructured subsets: the corporate reporting case

**DOI:** 10.1038/s41598-019-47490-0

**Published:** 2019-07-30

**Authors:** Eduardo Viegas, Hayato Goto, Misako Takayasu, Hideki Takayasu, Henrik Jeldtoft Jensen

**Affiliations:** 1 Centre for Complexity Science and Department of Mathematics, Imperial College London, SW7 2AZ London, United Kingdom; 2 Institute of Innovative Research, Tokyo Institute of Technology, 4259, Nagatsuta-cho, Yokohama, 226-8502 Japan; 3 Sony Computer Science Laboratories, 3-14-13, Higashi-Gotanda, Shinagawa-ku, Tokyo, 141-0022 Japan; 4 Department of Mathematical and Computing Sciences, School of Computing, Tokyo Institute of Technology, 4259, Nagatsuta-cho, Yokohama, 226-8502 Japan

**Keywords:** Applied mathematics, Complex networks, Nonlinear phenomena

## Abstract

The analysis of interfirm business transaction networks provides invaluable insight into the trading dynamics and economic structure of countries. However, there is a general scarcity of data available recording real, accurate and extensive information for these types of networks. As a result, and in common with other types of network studies - such as protein interactions for instance - research tends to rely on partial and incomplete datasets, i.e. subsets, with less certain conclusions. Here, we make use of unstructured financial and corporate reporting data in Japan as the base source to construct a financial reporting network, which is then compared and contrasted to the wider real business transaction network. The comparative analysis between these two rich datasets - the proxy, partially derived network and the real, complete network at macro as well as local structural levels - provides an enhanced understanding of the non trivial relationships between partial sampled subsets and fully formed networks. Furthermore, we present an elemental agent based pruning algorithm that reconciles and preserves key structural differences between these two networks, which may serve as an embryonic generic framework of potentially wider use to network research, enabling enhanced extrapolation of conclusions from partial data or subsets.

## Introduction

Financial reporting and corporate credit analysis are key publicly available information sources used by external stakeholders to form a view on potential risks, investment opportunities and creditworthiness of companies^1,2^. These reports are generally produced through a combination of automated data sourcing processes and a significant level of manual manipulation and editing. In addition, individuals carrying out such work tend to have their tasks restricted to either a specific company or limited number of companies within a given economic sector. The existence of such complex and non homogenous processes, carried out in silos by distinct individuals, leads to the generation of written reports that are extremely varied in terms of structure, content and form^3,4^. As a result, a systematic and standardised approach to source structured data from these reports is a near impossible task, even at research analyst, company provider or type of business level.

However, certain descriptive features - such as key clients, suppliers and banking relationships - are essential information to any corporation. Therefore, these will be commonly part of the content in any of these reports, albeit in potentially very distinct locations. By relying of these commonalities, it is possible to construct a generic network of relationships among companies solely based on the identification of company names within each individual company reports, and therefore creating a relationship between citing and cited companies. In this research, we define the network arising from such relationships as the ‘Financial Report Network’, or FRn. Here, the source node is the reporting company (i.e. the one being analysed or reporting on), whereas the target node relates to the companies cited within such report. From an edge - i.e. connection between two nodes - conceptual perspective, FRn records relationships between cited and citing companies.

Since most direct relationships between two firms involve some form of ‘business transaction’ (i.e. the exchange of goods or services in return to cash receipts and payments)^5^, FRn reflects a subset, i.e. some sort of a partial, proxy structure of the complete, fully formed ‘Interfirm Business Transaction Network’ or IBTn.

Firstly, FRn is build by automated text scanning of more than 3 million files within the first database, and applying a filtering method to eliminate false relationships. This is followed by a comparing and contrasting data analysis of the FRn to the actual, real IBTn^6,7^, where structural similarities and differences between these networks are highlighted from both macro and local levels. We then move on to develop an elemental agent based pruning algorithm that reduces the much larger IBTn to a subset that is similar to the FRn both in terms of size and certain structural features.

By following the above steps, we address a specific as well as a generic motivation to this research. From a narrow perspective, we intend to draw conclusions whether the use of unstructured financial reporting data can be used by researchers as well as market agents as the basis to generate a synthetic IBTn where data for the latter is not available.

Distinctly from Japan, other countries do not benefit from years of data collection work from private firms that enable direct access to the real ‘Interfirm Business Transaction Network’ or IBTn. Therefore, a method that enables to generation of a structurally similar IBTn can provide significant benefits to researchers and analysts providing significant support on the study of the dynamics of the trading networks within a selected country, or an economic block, for example.

From a wider perspective, we propose an embryonic generic framework that provides further insight into the effects and potential implications of incomplete and noisy network data, an important topic to networks in general, such as biological and social networks^8,9^. Data collection for these networks tend to be carried out partially, through non-homogenous processes, with contributions added over time from a number of different sources, distinct researches and varied methods. Essentially, these processes in aggregate may be seen as part of incrementally evolving dynamics which are conditioned by each earlier collection exercise. From such perspective, it is possible to regard financial reporting data generation as a basic evolutionary process that may be not too distinct from those of other networks.

Previous works on the real trade Japanese network^6,10–12^ are centered on the system dynamics evolutionary aspects of the network. In contrast, this research is more preoccupied with the generation and formation of networks from large scale unstructured data^13^, and the interaction between complete versus partial - proxy - network datasets and subsets^14,15^. Furthermore, the work is also interested in comparing the results of high dimensional space measurement methods^16,17^ to those of other more traditional network techniques when applied to large networks^18^.

## Results

Our results are presented is four distinct sections in tandem with the structure described within the Introduction section above, namely: (a) the construction of the ‘Financial Report Network’ (FRn), (b) the comparative network data analysis, (c) the agent based pruning algorithm and (d) network comparison of high dimensional space. Whereas specific inferences are presented within this section, broader conclusions are included within next section, ‘Conclusions and Discussion’.

### Formation of the financial report network

As described, FRn is solely built upon automated electronic scanning of all company names within the financial reporting and corporate credit reports database. Every instance where another company name is found within a given report, a relationships between citing (i.e. reporting) and cited companies is recorded as an edge within the network.

**Figure 1:**
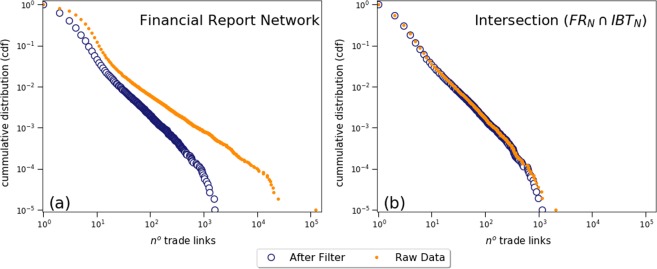
Filtering false edges from the Financial Report Network. The plot (**a**) on the left shows the cumulative distribution of companies (i.e. by number of trade links) for FRn in 2018 on a comparative basis, i.e. before and after application of the filtering method. A similar comparison is made at the plot (**b**) on the right for the intersection of edges between FRn and IBTn.

**Figure 2:**
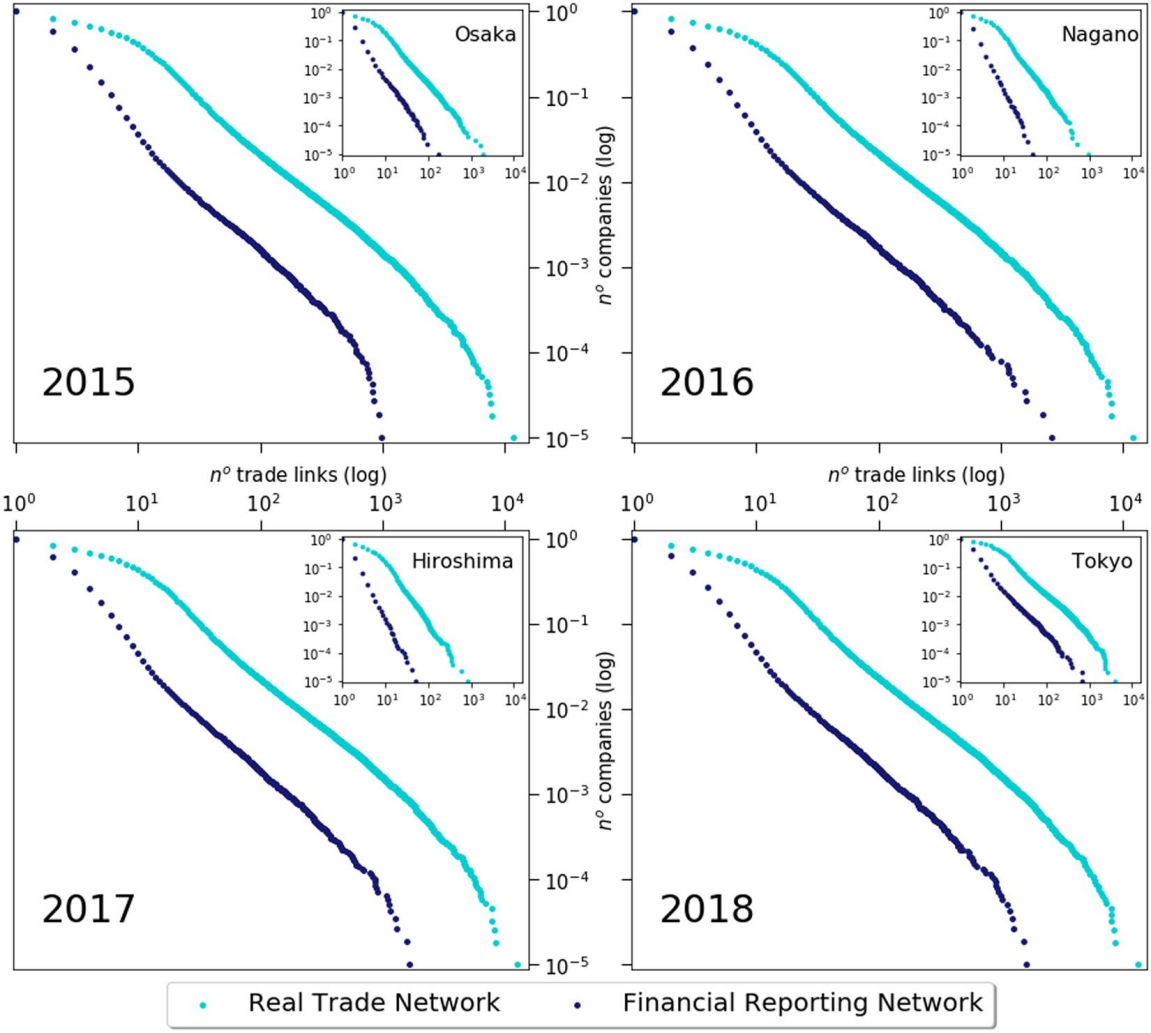
Cumulative degree distribution of companies within Japan and selected prefectures. Each main plot, drawn on logarithm scale, shows the cumulative distribution of companies in Japan as a function of the number of trade links (edges) for each year between 2015 to 2018. The insets provide similar information, but data is restricted to companies and transactions endogenous to the selected prefectures. The blue dots record FRn, whereas IBTn is represented by the turquoise dots.

Such data mining process, however, may generate incorrect, or false, edges, due to generic names being wrongly interpreted as a company names (i.e. nodes) as described in details within the Methods section. Therefore, such edges are largely concentrated within very few nodes within essentially predictable names. Unsurprisingly, the effect is substantially pronounced at tail end of a node degree distribution. This effect can be clearly observed by the plots within Fig. 1. The plot (a) shows the orange dots - representing the unfiltered, raw FRn network - result in very large numbers, and produce a slope which is not consistent with that of the intersection with the real trade network, IBTn, as observed within the plot (b). Once the filtering procedure is applied, however, FRn produces a slope consistent with IBTn, as observed in Fig. 2. Most importantly, the overall effectiveness of the filtering procedure is substantiated by the fact that the cumulative degree distributions resulting from the intersection of edges between FRn and IBTn is essentially and substantially preserved before and after filtering, as seen within the right side plot. Effectively, this means that very few known existing nodes are incorrectly removed during the process. Whereas only the 2018 results are presented here, similar results were obtained for the years 2015, 2016 and 2017.

### Comparative network data analysis

The cumulative degree distributions of firms as a function of their respective number of trading links, are set out for both networks, FRn and IBTn, in Fig. 2. Each large frame shows the link distributions among all companies within Japan for a given calendar year. By contrast, each inset relates exclusively to link distributions amidst companies within a selected prefecture.

It is noticeable that the slope coefficient of circa −1.5^7^ is essentially similar for both networks across all studied years. However, whereas IBTn maintains its slope at prefecture level, and therefore indicating a self-similar structure, FRn descends into a steeper grade for smaller prefectures. Such difference in behaviour can be attributed to the small size effect, since the number and size of firms within these prefectures are much smaller.

Whereas on a macroscopic, aggregated degree distribution level, both networks are very similar, upon other measurements, a more nuanced picture exists, and some differences start to emerge at structural level.

**Figure 3:**
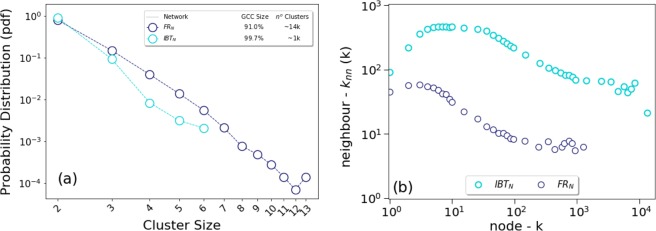
(**a**) Probability distribution function of clusters and (**b**) Neighbourhood connectivity of nodes. Plot (**a**) shows the probability distribution function of the nodes (other than the Giant Component Node, GCC) for FRn and IBTn networks. The legend inset contains a simple table with key metrics related to each of the relevant network. Plot (**b**) contains histograms of average degrees of nodes (under logarithm binning of the x-axis values) for FRn and IBTn networks plotted against the respective average degrees of all linked neighbours, y-axis.

Firstly, as observed in the turquoise and blue circles within plot (a) in Fig. 3, whereas a giant connected component (‘GCC’)^19^ is observed in both networks, IBTn is almost fully connected with 99.7 per cent of the nodes within such component, all all other clusters containing no more than five nodes. In contrast, FRn is much more fragmented, containing around 19 thousand isolated clusters, with the GCC representing circa 91 per cent of the total nodes. Both networks, however, present similar power law scaling factor of around −3.2 for their respective cluster distributions.

Secondly, at a local neighbourhood level, the degree correlation between a node and the average of its neighbours^15^ is much higher for IBTn when compared to FRn as shown in plot (b), Fig. 3.

### Agent based pruning algorithm

Through the pruning algorithm described in the Methods section, we have carried out a total of 100 individual simulations where IBTn is trimmed down into a subset with the number of edges equivalent to that of FRn.

**Figure 4:**
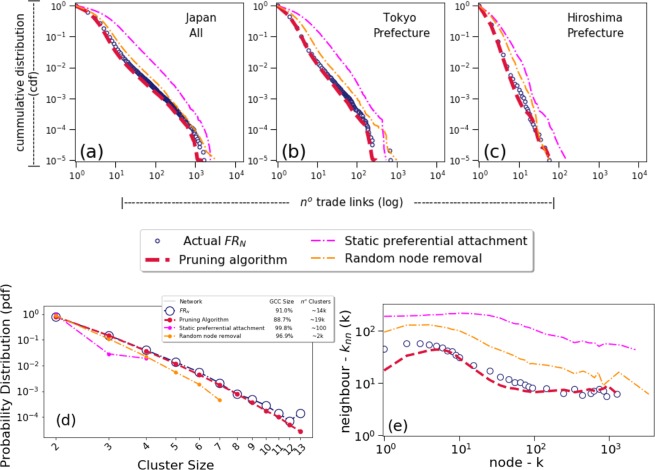
Comparison of results between the real *FR*_*N*_, pruning algorithm and alternative statistical methods at (**a**–**c**) macro level, degree distribution, (**d**) cluster and (**e**) local, connectivity levels. Plots (**a**–**c**), drawn on logarithm scale, shows the comparison between the actual and simulated results of the cumulative distribution function of trade links within the Financial Reporting Network (*FR*_*N*_) following the application of the pruning algorithm and other methods for all companies in Japan, Tokyo and Hiroshima prefectures only. Plot (**d**) corresponds to the equivalent comparison for the probability distribution function of the nodes (other than the Giant Component Node, GCC). whereas plot (**e**) contains histograms of average degrees of nodes plotted against the respective average degrees of all linked neighbours, y-axis. All plots are represented by the average results of 100 simulations for the pruning algorithm.

From a macroscopic degree distribution perspective, the resulting trimmed network is substantially similar to FRn, both in total and at fractal prefecture level. Figure 4 shows the average results of all 100 simulations for 2018, where: (a) relates to trade between all companies within Japan, (b) trades between companies located in Tokyo prefecture only, and (c) trades within Hiroshima only (i.e. combination of a large and a small prefecture). Analysis for other prefectures, as well as for the years 2015, 2016 and 2017, yield similar results. Here, we highlight the existence of two dots within the tail of the Tokyo distribution - plot (b) - that do not fit the overall simulation. Under granular inspection, we were able to verify that these relate to two specific large companies in Japan that have unusual concentration of reporting to companies within the Tokyo prefecture, and therefore, they can be regarded as outliers that cannot be specifically captured under a generic algorithm.

In addition, plot (d) - and metrics within the inset - shows that the resulting pruning subset also favourably compares to FRn when these networks are analysed from a cluster size distribution and giant component (i.e. the largest cluster) perspective. Whereas IBTn tends to effectively equate to a single large component, its subset (the Prunned IBTn) and FRn are very fragmented. This type of fragmentation seems to be similar to those observed within a number of research related to protein networks, and are typical of subsets of larger networks^14,15^.

At a more micro, local node interaction neighbourhood connectivity level, the resulting Prunned IBTn also provides results in line with structural features of FRn. This can be clearly observed in plot (e) of Fig. 4 where the node degrees k are plotted against the average degrees of their linked neighbours *k*_*nm*_(*k*). Agreement is particularly high at larger node degrees, whereas very small degrees (i.e. less than 4 edges) yielded slightly higher deviation.

The pruning algorithm also provides better performance when compared to two other more traditional statistical methods, namely: the random node removal process and the static preferential attachment (see Methods section). Within the former, whereas the fitting at macro, degree distribution level provides reasonably good results - as shown by the degree correlation plot on Fig. 4(e) - the procedure fails to capture the essential dynamics of local interaction levels, with way too many connections between smaller nodes to larger ones being preserved. In contrast, the static preferential attachment method - i.e. one solely based on information from a single node but without taking into account the overall changes to the composition of all other nodes - provides unfavourable fittings, albeit preserving the basic power law slope.

It is also worthy emphasising that if we were to select a source node in accordance with the cumulative advantage mechanism described in the Methods section (Network pruning algorithm) but randomly select a target node, the whole Prunned IBTn curve (in red) would have shifted significantly up, and therefore no longer in agreement with FRn. The degree distribution at macro level shown in plot (a) would, however, remain substantially similar.

In summary, the importance of a more generalised, evolutionary, preferential attachment method is highlighted when the results of the pruning algorithm are evaluated in tandem with those of other statistically based network removal methods, as shown in all frames within Fig. 4. Indeed, the intrinsic information from a single node, together with the composition of all other nodes, play a combined role in the selection process. This reflects a similar feature found in Jensen’s tangle nature model^20^, where the fitness of a node, or individual, is not solely based on its own features but also on the distribution of other individuals within a given system.

### Network comparison on high dimensional space

The previous section makes use of typical network theory measurements to compare the results of different methods^18^, both from a macro as well as local level interaction perspective. Here, we further extend our analysis to assess and evaluate the similarities between the actual *FR*_*N*_ and synthetic networks inspired by graph theory^16,17^ principles, based on the general, high dimensional, structures of graphs’ - or networks’ - continuous eigensepectrum distributions^21,22^.

**Figure 5:**
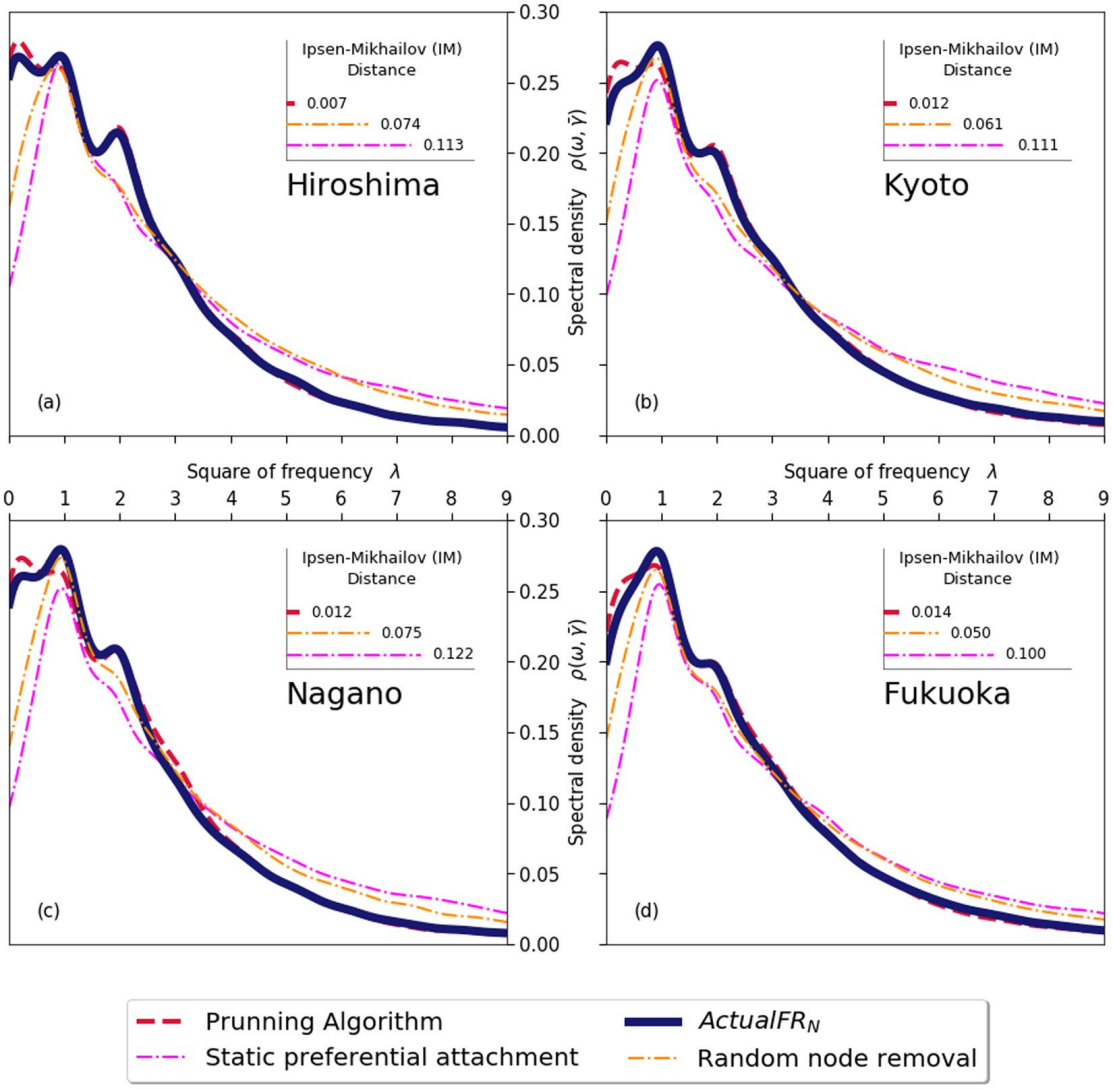
Lorentzian distribution of the Laplacian spectra and respective Ipsen-Mikhailov distances between the actual *FR*_*N*_ and distinct pruning methods. Each graph provides the spectral densities of the normalised Lorentzian distribution of the eigenvalues for the actual Financial Reporting Network (*FR*_*N*_) and comparative pruning methodologies for (**a**) Hiroshima, (**b**) Kyoto, (**c**) Nagano and (**d**) Fukuoka prefectures. The inset within each frame is a summarised bar chart for the Ipsen-Mikhailov distance, measured by the differences between the actual *FR*_*N*_ and the selected pruning method.

In Fig. 5 it is possible to observe broadly similar shapes with respect to the derived Lorentzian distribution of the Laplacian spectra of the eigenvalues related to the actual and simulated networks of four middle sized prefectures (Hiroshima, Kyoto, Nagano and Fukuoka). However, it is clearly noticeable that the pruning algorithm is able to replicate the tail ends as well as the peaks in a much enhanced manner when compared to the other methods - i.e statistical methods solely based on random removal or the preferential attachment method without evolutionary dynamics. The better fitting is also clearly reflected by the calculation of the Ipsen-Mihkalov distance^23^ between each of the synthetically derived network and the actual *FR*_*N*_ as shown by the insets within each graph.

We note here that we have limited our high dimensional analysis to prefecture level data - which are large datasets in isolation - due to the computational challenges associated with the calculation of all eigenvalues of large networks^24^. Indeed, research to date on network measurements and comparisons of high dimensional space methods - such as the Ipsen-Mihkalov distance^23^ - tend to be restricted to much smaller networks. Within this context, the consistency our results when applied to large networks - and compared to other more traditional network methods - provide further insight into to the potential application of high dimensional space methods as network comparison tools.

## Conclusions and Discussion

The result of this research indicate that it is feasibly possible to derive a generically and structurally representative interfirm business transaction network solely based on from unstructured data from financial and corporate credit analysis documents.

Although the resulting synthetic proxy network is only a subset of the wider (real) network, it contains some of its key features, and therefore provides a valuable insight into the business dynamics of a country via a subset when data is not availble.

However, accurate and informative extrapolation of larger synthetic networks in general, or related reversing process (i.e. sampling), is only likely to be successfully achieved if essential features underpinning the dynamics and generation of the networks in study are well understood and modelled accordingly.

This study provides further support to the fact that straight forward statistical selections and random methods are unlikely to yield fully representative results for a broader range of network features, both at macroscopic as well as at localised, neighbourhood based interaction levels^8,25^. As a result, we believe our findings may be helpful to inspire new methods by researchers working on social and biological networks that need to rely on samples, incomplete and/or noisy network datasets^26^.

Moreover, our results indicate that the broader use on large networks of the Lorentzian distribution of the Laplacian spectra and related Ipsen-Mikhailov distances provide useful complement to traditional network measurements. Future work on pairing and adjusting such approach with computational methods that estimate eingenvalues of very large networks^24^ may enable the development of mathematically richer comparison measurements and methods.

More specifically to the existing networks in study, the current method can be potentially enhanced by making further use of scaling relationships and correlations with regards to geographical distance, company size, and economic sector. This can be combined with community detection and indirect relationships methods^13^ which would significantly reduce the level of uncertainty of edges generation (in case of a growth model) or removal (in case of sampling). However, one needs to be cautioned to the fact that the formation and construction of the Financial Report Network is highly dependent on obtaining reports and documents of smaller and middle sized entities, since there is no reciprocity on the reporting from large entities. Such information is not always to the availability of the general public, given that financial reporting and coverage of credit analysis tends to be limited to larger entities and corporations.

## Methods

### Construction of the networks

This research analyses two rich datasets provided by Teikoku Databank Ltd., namely: (a) the financial reporting and corporate credit report; and (b) the business trade network database. The information within each database are sourced independently from each other, containing records - electronically stored since 1974 - for over 800,000 companies in Japan. Here, we make use of a four year period between 2015 to 2018.

The construction of the Interfirm Business Trade Network, IBTn, is a straight forward process, since the database contains an ID number for each company (which can be mapped to a similar government company registration number) together with the ID number of its customers and suppliers^10,11^. Therefore, each company maps to a node within the network, with edges representing the customer/supplier relationship. Edges can be directed based on the flow of monies or - in reverse - by the delivery of goods and services. However, the direction of the edges are not relevant to the scope of this reserach.

In contrast, the Financial Reporting Network, FRn, is generated by a data mining process of financial and corporate credit reports. Within this process each company represented by a node with a specific unique ID, with the source node being the reporting company (i.e. the one being analysed or reported on), whereas the target node relates to the companies cited within such report, and mapped to their respective unique IDs. Therefore, edges essentially capture the relationships between cited and citing companies. Given that financial reporting standards and requirements state that key activities and relationships between customers and suppliers require specific disclosure^27^, the citation links will encompass a fraction of - and be substantially equivalent to - the relationships recorded within IBTn from an undirected perspective, and therefore enabling direct comparison from an undirected perspective.

At very basic level, the formation of FRn is akin to that of citation networks^28,29^. However, fundamental differences exists such since data is fully unstructured, and there is no time ordering of vertices and direction.

### Filtering process

The database underpinning IBTn is comprehensive and consistent. It has also been extensively used in a number of research publications, and therefore it requires no cleansing. In contrast, FRn was generated by automated scanning processes of unstructured data - i.e. by finding names of companies within financial reports. Such computing processes inevitably lead to some degree of unreliable data. As a result, filtering and cleansing procedures are required before any rigorous analysis is carried out.

Our filtering method is based upon the existing scaling relationships within business transaction networks. Previous studies^5,6,10^ show that the number of trading links within the IBTn is highly correlated to other quantities such as number of employees, total assets and income. Therefore, it is only natural to make use of such relationships to remove incorrect data within FRn.

The proposed filtering process is not to be applied universally to all types of networks. However, it can be generically applied if the networks in study are subject to scalling relationships - such as merger networks^30^, food webs^31^, metabolism^32^, etc. Scalling is a common feature of a number of biological and social networks^33^. This is a simple approach to ensure that the false relationships are substantially eliminated within FRn through automated means. We emphasise, however, that the filtering process is not central to the research, and other more advanced methods - as well as manual methods - could also be used. In any case, these are likely to have minimal impact on the structure of the network.

**Figure 6:**
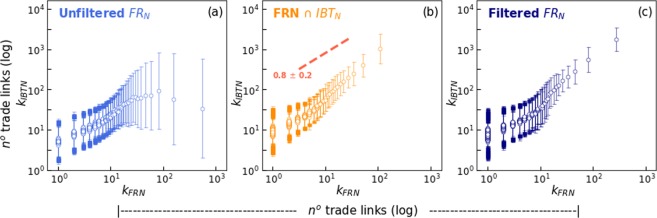
Scalling relationship of trade links. The circles within each plot represent the pairing of the number of trade links of FRn (x-axis), and the average number of trade links for IBTn (y-axis). The error bars indicate the lower and upper boundaries of two standard deviations, 2*σ*^2^, from the average IBTn. The plot on the left (**a**) records unfiltered FRn data, whereas the right plot (**c**) represents FRn after filtering. The centre plot (**b**) shown the intersection of both IBTn and FRn datasets, whereas the red line indicates the scalling factor.

Figure 6 illustrates the logic. Whereas the scaling relationship is maintained at lower number of links on plot (a), it starts breaking down for higher values. This is in clear contrast to the data shown within plot (b), i.e. the intersection set, where the scalling relationship is continuous. It follows that links that significantly deviate from scalling, beyond two standard deviations, are deleted. The results on plot (c), read in conjunction with Fig. 1(b) show the elimination of outliers, but the preservation of the known data points.

On a granular inspection, we were able to verify that significant part of the breakdown of the relationships were due to very small companies that had generic names such as ‘China’, ‘Tokyu’, ‘Imperial’ that are also referred in financial reports with a total different meaning (‘China’, for example being a reference to the country rather than the company).

The granular inspection that validates the eliminations, together with the preservation of the known data points as shown in Fig. 1(b) provide appropriate comfort and assurance that the filtering process is an adequate method to eliminate such incorrect data without introducing any unintended bias.

### Network pruning algorithm

The pruning algorithm is essentially inspired by Yule’s classical workings^34^ on evolution of species and distributions, Price’s cumulative advantage theory^35^, and Barabasi’s application of the preferential attachment concept to networks^34,36^.

However, distinctively from the classical preferential attachment equation where weightings are solely dependent upon the intrinsic characteristic of each node^18,36^, we introduce additional evolutionary dynamics where weightings are impacted - and adjusted by - the composition of other participating nodes. Such approach is inspired by Jensen’s tangled nature model framework^20^, where the behaviour or each node is not solely dependent on its individual fitness but shaped in accordance with the other nodes within the environment. Furthermore, inspiration and measuring methods are also drawn from Ipsen-Mikhailov’s evolutionary framework for reconstruction of networks^23^.

In order to demonstrate and highlight the importance of both dynamics acting together, namely, the preferential attachment mechanism as well as the evolutionary mechanism, we generate and compare the results of three different pruning methods. Firstly, we adopt the statistical random node removal method as described by Stumpf^9,37^. Secondly, we modify the random selection by implementing the classic, static, preferential attachment process^36^. Essentially the method adds weights to the probabilities of selection within the random process. Lastly, we adopt our pruning algorithm where these weightings are further modified by the composition of all the nodes within the system at the time of removal.

When constructing the algorithm, we address two specific issues. The first matter is applicable to all networks in general: a random sampling of a scale-free network does not lead to similar scale-free distributions for the subset^37^. In contrast, the second matter relates to specific structural differences between the networks (i.e. the full IBTn set and the FRn subset). Within IBTn, the in and out degree distributions are balanced, resulting in equal power law probability distributions. In contrast, the FRn is unbalance since the in degree distributions follow similar power law behaviour, but not the out degrees. This means that whereas smaller and middle size companies tend to cite most relationships, large companies provide very limited citation information. Essentially this leads to an observed rich-club phenomena within IBTn which is not present in FRn.

In order to address these two issues, the pruning algorithm is built in two stages, corresponding to sequencing the selection of two (connected) nodes, source and target, instead of directly selecting an edge to be removed.

#### The algorithm flow

The algorithm follows a straight flow where an edge is removed by selecting the source, and subsequently the target, nodes by the normalising the probabilities of all nodes available for selection:

1 $$Pn(K)=\frac{P(k)}{{\sum }_{i=1}^{n}P(i)},{\rm{w}}{\rm{h}}{\rm{e}}{\rm{r}}{\rm{e}}\{\begin{array}{c}{\rm{P}}{\rm{n}}({\rm{K}})\,{\rm{i}}{\rm{s}}\,{\rm{t}}{\rm{h}}{\rm{e}}\,{\rm{n}}{\rm{o}}{\rm{r}}{\rm{m}}{\rm{a}}{\rm{l}}{\rm{i}}{\rm{s}}{\rm{e}}{\rm{d}}\,{\rm{p}}{\rm{r}}{\rm{o}}{\rm{b}}{\rm{a}}{\rm{b}}{\rm{i}}{\rm{l}}{\rm{i}}{\rm{t}}{\rm{y}}\,{\rm{o}}{\rm{f}}\,{\rm{P}}({\rm{k}})\\ {\rm{P}}({\rm{k}})\,{\rm{i}}{\rm{s}}\,{\rm{t}}{\rm{h}}{\rm{e}}\,{\rm{p}}{\rm{r}}{\rm{o}}{\rm{b}}{\rm{a}}{\rm{b}}{\rm{i}}{\rm{l}}{\rm{i}}{\rm{t}}{\rm{y}}\,{\rm{f}}{\rm{o}}{\rm{r}}\,{\rm{e}}{\rm{a}}{\rm{c}}{\rm{h}}\,{\rm{n}}{\rm{o}}{\rm{d}}{\rm{e}}\,{\rm{c}}{\rm{a}}{\rm{l}}{\rm{c}}{\rm{u}}{\rm{l}}{\rm{a}}{\rm{t}}{\rm{e}}{\rm{d}}\,{\rm{i}}{\rm{n}}\,{\rm{a}}{\rm{c}}{\rm{c}}{\rm{o}}{\rm{r}}{\rm{d}}{\rm{a}}{\rm{n}}{\rm{c}}{\rm{e}}\,{\rm{t}}{\rm{o}}\,{\rm{e}}{\rm{q}}{\rm{u}}{\rm{a}}{\rm{t}}{\rm{i}}{\rm{o}}{\rm{n}}\,2\,{\rm{b}}{\rm{e}}{\rm{l}}{\rm{o}}{\rm{w}}\end{array}$$

with the denominator consisting of all nodes when selecting the source, in contrast to all nodes connected to the source when selecting the target. The edge linking source and target is then removed, and the degree of the nodes updated. This process is followed until the total number of remaining edges equals to the total number of edges within *k*_*FRN*_.

#### The probability of selection of nodes and edges

Each node is given a probability of selection:

2 $$S(k)=1-{e}^{-\frac{(k+1)}{M}},{\rm{where}}\{\begin{array}{l}{\rm{S}}({\rm{k}})\,{\rm{is}}\,{\rm{the}}\,{\rm{probability}}\,{\rm{of}}\,{\rm{selection}}\,{\rm{of}}\,{\rm{node}}\,{\rm{with}}\,{\rm{degree}}\,{\rm{k}}\\ {\rm{M}}\,{\rm{is}}\,{\rm{the}}\,{\rm{middle}}\,{\rm{of}}\,{\rm{the}}\,{\rm{zipf}}\,{\rm{rank}}\,{\rm{of}}\,{\rm{degrees}}\,{\rm{K}}\,{\rm{available}}\,{\rm{for}}\,{\rm{selection}}\end{array}$$

The above probability of node selection equates to an approximation of the cumulative mass function of a geometric distribution, where *S*(*k*) = *P*(*K* < *k*). Therefore, it can be written as:

3 $$P(K < k)=1-{(1-\frac{1}{M})}^{k+1},{\rm{where}}\{\begin{array}{l}{\rm{P}}({\rm{K}} < {\rm{k}})\,{\rm{is}}\,{\rm{the}}\,{\rm{cumulative}}\,{\rm{number}}\,{\rm{of}}\,{\rm{successes}}\,{\rm{for}}\,{\rm{the}}\,{\rm{set}}\,{\rm{K}}=\{0,1,2,\mathrm{..},{\rm{k}}\}\\ \llcorner \,{\rm{before}}\,{\rm{first}}\,{\rm{failure}},({\rm{i}}.{\rm{e}}\,{\rm{K}}={\rm{k}})\\ \frac{1}{{M}}{\rm{represents}}\,{\rm{generic}}\,{\rm{probability}}\,{\rm{of}}\,{\rm{addition}}\,{\rm{of}}\,{\rm{an}}\,{\rm{edge}}\,{\rm{to}}\,{\rm{node}}\,{\rm{of}}\,{\rm{degree}}\,{\rm{k}}\end{array}$$

Here, we elaborate on the choice of the functional form of Eq. 2 and the equivalent Eq. 3.

Firstly, the pruning mechanism is not time dependent, since both networks, set and subset, are frozen at time ‘t’. In this case the geometric distribution is appropriate as we are only preoccupied with the number of ‘﻿success’ events (i.e. addition of an edge), and the order of their removal. Therefore, ‘failures’ can be ignored. The pruning algorithm removes an edge at every single step, and therefore no corresponding ‘﻿f﻿ailure’ events are to be selected.

Secondly, the cumulative mass function is used since every single element of the set K < k needs to be removed in order for all ‘﻿success’ events to be reversed.

Thirdly, *k* and *M* can be regarded as the endogenous and exogenous factors affecting a probability of a node to be selected. Here, *k* represents the fact that a node with a large number of edges - in isolation - is intrinsically more likely to add further edges (i.e. the preferential attachment, or cumulative advantage principle). In contrast, *M* represents the effect of the overall environment on each node, where the more diverse the population (in terms of numbers of edges), the higher tends to be the general probability of origination of new edges. These two elements bear a conceptual resemblance to the reproduction dynamics of Jensen’s Tangled Nature model^20^ where the ability of an individual to reproduce is dependent upon (a) its strength of interactions and (b) the carrying capacity of the environment.

### Network comparison and the ipsen-mikhailov distance

Extensive research exists in relation to network measures and comparison methods. Essentially, the selection of methods are fundamentally dependent on the size and structure of the network, the features that are important for the selected research, as well as the researcher preferences.

Our method makes use of three traditional distribution measures derived from network theory^6,18^ that are applicable to very large networks, namely: degree distributions and cluster and giant component distributions to evaluate global features as well as the average degree correlations to assess local level interactions.

Furthermore, inspired by graph theory and statistical physics concepts, we also make use of the Lorentzian distribution of the Laplacian spectra^16,17^ and related Ipsen-Mikhailov^23^ distances and apply to large subsections, or prefecture levels, of our network.

The Ipsen-Mikhailov metric is originally derived from modelling the frequencies of vibrating strings connected to a network system of N molecules. The vibrational strings within such system can be derived from the eigenvalues of the Laplacian matrix of such network, where the spectral density of the is the sum of Lorentz distributions, defined as:

4 $$\rho (\omega ,\gamma )=K\sum _{i=1}^{N-1}\,P\frac{\gamma }{{(\omega -{\omega }_{i})}^{2}+{\gamma }^{2}},{\rm{w}}{\rm{h}}{\rm{e}}{\rm{r}}{\rm{e}}\{\begin{array}{c}{\omega }_{{\rm{i}}}\,{\rm{i}}{\rm{s}}\,{\rm{t}}{\rm{h}}{\rm{e}}\,{\rm{v}}{\rm{i}}{\rm{b}}{\rm{r}}{\rm{a}}{\rm{t}}{\rm{i}}{\rm{o}}{\rm{n}}{\rm{a}}{\rm{l}}\,{\rm{f}}{\rm{r}}{\rm{e}}{\rm{q}}{\rm{u}}{\rm{e}}{\rm{n}}{\rm{c}}{\rm{y}},{\rm{g}}{\rm{i}}{\rm{v}}{\rm{e}}{\rm{n}}\,{\rm{b}}{\rm{y}}\,{\rm{t}}{\rm{h}}{\rm{e}}\,{\rm{s}}{\rm{q}}{\rm{u}}{\rm{a}}{\rm{r}}{\rm{e}}\,{\rm{r}}{\rm{o}}{\rm{o}}{\rm{t}}\,{\rm{o}}{\rm{f}}\,{\rm{t}}{\rm{h}}{\rm{e}}\,{\rm{e}}{\rm{i}}{\rm{g}}{\rm{e}}{\rm{n}}{\rm{v}}{\rm{a}}{\rm{l}}{\rm{u}}{\rm{e}}{\rm{s}},\lambda \\ {\rm{K}}\,{\rm{i}}{\rm{s}}\,{\rm{a}}\,{\rm{s}}{\rm{c}}{\rm{a}}{\rm{l}}{\rm{l}}{\rm{i}}{\rm{n}}{\rm{g}}\,{\rm{f}}{\rm{a}}{\rm{c}}{\rm{t}}{\rm{o}}{\rm{r}},{\rm{a}}{\rm{n}}{\rm{d}}\\ \gamma \,{\rm{t}}{\rm{h}}{\rm{e}}\,{\rm{p}}{\rm{a}}{\rm{r}}{\rm{a}}{\rm{m}}{\rm{e}}{\rm{t}}{\rm{e}}{\rm{r}}\,{\rm{s}}{\rm{p}}{\rm{e}}{\rm{c}}{\rm{i}}{\rm{f}}{\rm{y}}{\rm{i}}{\rm{n}}{\rm{g}}\,{\rm{w}}{\rm{i}}{\rm{d}}{\rm{t}}{\rm{h}},{\rm{w}}{\rm{i}}{\rm{t}}{\rm{h}}\,\bar{\gamma }\sim 0.478\,{\rm{f}}{\rm{o}}{\rm{r}}\,{\rm{l}}{\rm{a}}{\rm{r}}{\rm{g}}{\rm{e}}\,{\rm{N}}\end{array}$$

Once the spectral densities are generated for each distinct network, relative structural comparison is possible by overlaying the plotting of spectral densities as exemplified in Fig. 5. Differences between densities of two networks can be further synthesised into a single number, the Ipsen-Mikhailov distances (insets of Fig. 5) as follows:

5 $$IM({G}_{1},{G}_{2})=\sqrt{{\int }_{0}^{\infty }\,[{\rho }_{{G}_{1}}(\omega ,\bar{\gamma })-{\rho }_{{G}_{2}}(\omega ,\bar{\gamma })]{}^{2}\delta \omega },{\rm{where}}\{\begin{array}{l}{G}_{1}\,{\rm{and}}\,{G}_{2}\,{\rm{are}}\,{\rm{the}}\,{\rm{representation}}\,{\rm{of}}\,{\rm{two}}\,{\rm{networks}},\,{\rm{and}}\\ {\rho }_{{{\rm{G}}}_{1}}(\omega ,\bar{\gamma })\,{\rm{and}}\,{\rho }_{{{\rm{G}}}_{2}}(\omega ,\bar{\gamma })\,{\rm{their}}\,{\rm{respective}}\,{\rm{spectral}}\,{\rm{densities}}\end{array}$$

Whether our networks in study can be abstractly equated to a system of vibrating strings is a conceptual matter for debate. The application of the method, however, provides results that are very consistent to those obtained by traditional distribution measures, which empirically indicates some merit.

## References

[1] Akins B (2018). Financial reporting quality and uncertainty about credit risk among ratings agencies. Account. Rev..

[2] DeZoort FT, Wilkins A, Justice SE (2017). The effect of sme reporting framework and credit risk on lenders’ judgments and decisions. J. Account. Public Policy.

[3] Wojahn O, Geister S, Richter J (2015). The impact of analyst report complexity on trading decisions in an experimental setting. J. Behav. Exp. Finance.

[4] Fogarty T, Rogers R (2005). Financial analysts’ reports: an extended institutional theory evaluation. Accounting, Organ. Soc..

[5] Goto, H., Takayasu, H. & Takayasu, M. Estimating risk propagation between interacting firms on inter-firm complex network. *PLoS One* 12 (2017). 10.1371/journal.pone.0185712 PMC5626445 28972998

[6] Goto H, Viegas E, Jensen HJ, Takayasu H, Takayasu M (2017). Appearance of unstable monopoly state caused by selective and concentrative mergers in business networks. Sci. Reports.

[7] Kawamoto, H., Takayasu, H., Jensen, H. & Takayasu, M. Precise calculation of a bond percolation transition and survival rates of nodes in a complex network. *PLoS One* **10** (2015). 10.1371/journal.pone.0119979 PMC4401659 25885791

[8] Stumpf MPH, Wiuf C (2010). Incomplete and noisy network data as a percolation process. J. Royal Soc. Interface.

[9] Smith JA, Moody J (2013). Structural effects of network sampling coverage i: Nodes missing at random. Soc. Networks.

[10] Goto H, Viegas E, Jensen H, Takayasu H, Takayasu M (2018). Smoluchowski equation for networks: Merger induced intermittent giant node formation and degree gap. J. Stat. Phys..

[11] Miura, W., Takayasu, H. & Takayasu, M. Effect of coagulation of nodes in an evolving complex networks. *Phys. review letters* 108 (2012). 10.1103/PhysRevLett.108.168701 22680760

[12] Takayasu, M. *et al*. Massive economics data analysis by econophysics method-the case of companies’ network structure. *Behav. Ecol. Sociobiol*. 263–268 (2008).

[13] Al-Zaidy R, Fung BC, Youssef AM, Fortin F (2012). Mining criminal networks from unstructured text documents. Digit. Investig..

[14] Stumpf MPH, Thomas T (2006). Multi-model inference of network properties from incomplete data. J. Integr. Bioinforma..

[15] Lee, S. H., Pan-Jun, K. & Hawoong, J. Statistical properties of sampled networks. *Phys. Rev. E. Stat. Nonlinear, And Soft Matter Phys*. 73 (2006). 10.1103/PhysRevE.73.016102 16486211

[16] Donnat C, Holmes S (2018). Tracking network dynamics: A survey using graph distances. Ann. Appl. Stat..

[17] Jurman G, Visintainer R, Furlanello C (2010). An introduction to spectral distances in networks. Front. Artif. Intell. Appl..

[18] Newman, M. E. J. *Networks: an introduction* (Oxford University Press, 2010).

[19] Kitsak, M. *et al*. Stability of a giant connected component in a complex network. *Phys. review. E* **97** (2018). 10.1103/PhysRevE.97.012309 29448477

[20] Christensen K, DI Collobiano SA, Hall M, Jensen HJ (2002). Tangled Nature: A model of evolutionary ecology. J. Theor. Biol..

[21] Gu J, Hua B, Liu S (2015). Spectral distances on graphs. Discret. Appl. Math..

[22] Jurman, G., Visintainer, R., Filosi, M., Riccadonna, S. & Furlanello, C. The him global metric and kernel for network comparison and classification. *2015 IEEE Int. Conf. on Data Sci. Adv. Anal. (DSAA)* 1–10, 10.1109/DSAA.2015.7344816 (2015).

[23] Ipsen, M. & Mikhailov, A. S. Evolutionary reconstruction of networks. *Phys. review. E, Stat. nonlinear, soft matter physics* **66** (2002). 10.1103/PhysRevE.66.046109 12443261

[24] Sorensen DC (2002). Numerical methods for large eigenvalue problems. Acta Numer..

[25] Blagus N, Subelj L, Bajec M (2017). Empirical comparison of network sampling: How to choose the most appropriate method?. Phys. A: Stat. Mech. its Appl..

[26] Franks D, James R, Noble J, Ruxton G (2009). A foundation for developing a methodology for social network sampling. Behav. Ecol. Sociobiol..

[27] IASB. *International financial reporting standards (IFRS’s): including international accounting standards (IAS’s) and interpretations as at* (2018).

[28] Clough J. R., Gollings J., Loach T. V., Evans T. S. (2014). Transitive reduction of citation networks. Journal of Complex Networks.

[29] Goldberg SR, Anthony H, Evans TS (2015). Modelling citation networks. Scientometrics.

[30] Viegas, E., Cockburn, S. P., Jensen, H. J. & West, G. B. The dynamics of mergers and acquisitions: ancestry as the seminal determinant. *Proceedings. Math. Phys. Eng. Sci./The Royal Soc*. 470 (2014). 10.1098/rspa.2014.0370 PMC4197467 25383025

[31] Rossberg, A. *Food webs and biodiversity: foundations, models, data* (2013).

[32] West GB (1997). A general model for the origin allometric scaling laws in biology. Science.

[33] Daepp, M. I. G., Hamilton, M. J., West, G. B. & Bettencourt, L. M. A. The mortality of companies. *J. Royal Soc. Interface/Royal Soc*. 12 (2015). 10.1098/rsif.2015.0120 PMC4424689 25833247

[34] Yule GU (1925). A mathematical theory of evolution, based on the conclusions of dr. j. c. willis, f.r.s. Philos. Transactions Royal Soc. London. Ser. B, Containing Pap. a Biol. Character.

[35] Price DDS (1976). A general theory of bibliometric and other cumulative advantage processes. J. Am. Soc. for Inf. Sci..

[36] Barabási A-L, Albert R (1999). Emergence of scaling in random networks. Science.

[37] Stumpf M. P. H., Wiuf C., May R. M. (2005). Subnets of scale-free networks are not scale-free: Sampling properties of networks. Proceedings of the National Academy of Sciences.

